# Caesarean section and childhood obesity at age 3 years derived from the Japan Environment and Children’s Study

**DOI:** 10.1038/s41598-023-33653-7

**Published:** 2023-04-21

**Authors:** Shintaro Terashita, Taketoshi Yoshida, Kenta Matsumura, Takehiro Hatakeyama, Hidekuni Inadera, The Japan Environment and Children’s Study (JECS) Group, Michihiro Kamijima, Shin Yamazaki, Yukihiro Ohya, Reiko Kishi, Nobuo Yaegashi, Koichi Hashimoto, Chisato Mori, Shuichi Ito, Zentaro Yamagata, Hidekuni Inadera, Takeo Nakayama, Tomotaka Sobue, Masayuki Shima, Hiroshige Nakamura, Narufumi Suganuma, Koichi Kusuhara, Takahiko Katoh, Michihiro Kamijima

**Affiliations:** 1 Department of Pediatrics, Faculty of Medicine, University of Toyama, 2630 Sugitani, Toyama, 930-0194 Japan; 2 Division of Neonatology, Maternal and Perinatal Center, Toyama University Hospital, Toyama, Japan; 3 Department of Public Health, Faculty of Medicine, University of Toyama, Toyama, Japan; 4 Toyama Regional Center for JECS, University of Toyama, Toyama, Japan; 5 Graduate School of Medical Sciences, Department of Occupational and Environmental Health, Nagoya City University, 1 Kawasumi, Mizuho-Cho, Mizuho-Ku, Nagoya, Aichi 467-8601 Japan; 6 National Institute for Environmental Studies, Tsukuba, Japan; 7 National Center for Child Health and Development, Tokyo, Japan; 8 Hokkaido University, Sapporo, Japan; 9 Tohoku University, Sendai, Japan; 10 Fukushima Medical University, Fukushima, Japan; 11 Chiba University, Chiba, Japan; 12 Yokohama City University, Yokohama, Japan; 13 University of Yamanashi, Chuo, Japan; 14 Kyoto University, Kyoto, Japan; 15 Osaka University, Suita, Japan; 16 Hyogo College of Medicine, Nishinomiya, Japan; 17 Tottori University, Yonago, Japan; 18 Kochi University, Nankoku, Japan; 19 University of Occupational and Environmental Health, Kitakyushu, Japan; 20 Kumamoto University, Kumamoto, Japan

**Keywords:** Endocrine system and metabolic diseases, Health care, Paediatrics, Neonatology

## Abstract

Caesarean section (CS) birth is widely reported to be a risk factor for childhood obesity. Although susceptibility to childhood obesity is influenced by race and ethnicity, it is unclear whether this risk of childhood obesity with CS birth also applies in the Japanese population. We investigated the impact of CS birth on obesity at 3 years of age in Japanese children. We obtained data from 60,769 mother–toddler pairs in the Japan Environment and Children’s Study, a large-scale birth cohort study. Obesity was determined by body mass index measured at 3 years of age. Analysis revealed that 11,241 toddlers (18.5%) had a CS birth and that 4912 toddlers (8.1%) were obese. The adjusted risk ratio for obesity at 3 years of age when born by CS compared with vaginal delivery, estimated using inverse probability of treatment weighting, was 1.16 (95% confidence interval 1.08–1.25). These results suggest that CS birth modestly increases the risk of obesity at 3 years of age in Japanese children.

## Introduction

Childhood obesity is an important public health problem because it tends to carry over into adulthood and increases the risk of cardiovascular morbidity^1^. According to a meta-analysis of obesity prevalence studies^2^, children with obesity have a relative risk of adulthood obesity that is 5.21 times that of children who are not obese. Given that almost 90% of children who are obese at 3 years of age are obese in adolescence^3^, it is important to be able to predict early childhood obesity to avoid obesity later in life.

Childhood obesity is caused by a variety of factors. In recent years, many studies have reported that caesarean section (CS) birth modestly increases the risk of childhood obesity^4,5^. Although the mechanism by which CS birth increases obesity is unclear, one potential explanation is alteration of the gut microbiome. Infants born by CS have altered composition of the gut microbiome compared with those delivered by vaginal birth^6,7^ and there may be an association with childhood obesity^8^. Because the CS birth rate has increased worldwide^9^, there is raised concern that CS birth is a risk factor for obesity in children^10^.

Racial and ethnic background is also important when the risk of childhood obesity is being considered, with Asians having a 1.7 times higher prevalence of childhood obesity than White/European individuals^11^. Japan is an island nation located in East Asia and is composed almost entirely of a single ethnic group. The traditional Japanese lifestyle is considered less prone to obesity^12^, and a study comparing the prevalence of childhood obesity across the 35 Organisation for Economic Cooperation and Development countries revealed that Japan had the lowest prevalence^13^. However, while some reports have found that CS birth is a risk factor for childhood obesity in Asia^5,14^, none of the previous studies have examined whether CS birth could be a risk factor for childhood obesity specifically in the Japanese population. This is somewhat surprising considering that previous studies have revealed the association between CS and obesity in various races and ethnicities.

In this study, we therefore investigated whether CS birth is associated with obesity at 3 years of age in the Japanese population using a large-scale national birth cohort. We hypothesised that CS birth would be an independent risk factor for childhood obesity even in the Japanese population.

## Results

Of the 60,769 mother–toddler pairs analysed (Table 1), 31,036 toddlers (51.1%) were male and 11,241 toddlers (18.5%) had a CS birth. The proportion of preterm infants born before 37 weeks was higher in the CS birth group (n = 1140, 10.1%) than in the vaginal birth group (n = 1489, 3.0%), and the CS birth group had lower mean birth weight and height. The proportion of mothers older than 35 years during pregnancy was higher in the CS group (38.9% vs 25.6%), as was the proportion with a body mass index (BMI) ≥ 25 (n = 1748 [15.6%] vs n = 4181 [8.4%]). Mothers in the CS birth group also less commonly attended higher education and had a higher annual household income, and more of them had pregnancy complications, obstetric complications, a history of smoking, history of physical illness, and history of using assisted reproduction technology. There was little difference in alcohol consumption or parity (Table 1).

**Table 1:** Characteristics of the study population.

Variable	Yes (n = 11,241)	No (n = 49,528)
	n (%)	n (%)
Age during pregnancy, year		
< 25	581 (5.2)	4067 (8.2)
25 to < 30	2361 (21.0)	14,491 (29.3)
30 to < 35	3930 (35.0)	18,279 (36.9)
≥ 35	4369 (38.9)	12,691 (25.6)
Pre-pregnancy body mass index, kg/m^2^		
< 18.5	1425 (12.7)	8272 (16.7)
18.5 to < 25	8068 (71.8)	37,075 (74.9)
≥ 25	1748 (15.6)	4181 (8.4)
Highest education level, year		
≤ 12	3747 (33.3)	15,763 (31.8)
12 to < 16	4962 (44.1)	21,590 (43.6)
≥ 16	2532 (22.5)	12,175 (24.6)
Annual household income, million JPY		
< 4	4195 (37.3)	18,899 (38.2)
4 to < 6	3745 (33.3)	16,691 (33.7)
≥ 6	3301 (29.4)	13,938 (28.1)
Smoking history		
Never	6571 (58.5)	30,352 (61.3)
Former	4249 (37.8)	17,548 (35.4)
Current	421 (3.8)	1628 (3.3)
Alcohol intake		
Never	3737 (33.2)	16,712 (33.7)
Former	7226 (64.3)	31,453 (63.5)
Current	278 (2.5)	1363 (2.8)
Pregnancy complications		
No	8967 (79.8)	42,515 (85.8)
Yes	2274 (20.2)	7013 (14.2)
Obstetric complications		
No	5169 (46.0)	27,335 (55.2)
Yes	6072 (54.0)	22,193 (44.8)
History of any physical disease		
No	1555 (13.8)	8320 (16.8)
Yes	9686 (86.2)	41,208 (83.2)
Parity		
Primipara	5005 (44.5)	21,831 (44.1)
Multipara	6236 (55.5)	27,697 (55.9)
Use of assisted reproduction technologies		
No	10,503 (93.4)	48,188 (97.3)
Yes	738 (6.6)	1340 (2.7)
Child sex		
Male	5716 (50.9)	25,320 (51.1)
Female	5525 (49.2)	24,208 (48.9)
Birth term		
< 37 weeks	1140 (10.1)	1489 (3.0)
37 to < 42 weeks	10,042 (89.3)	47,947 (96.8)
≥ 42 week	59 (0.5)	92 (0.2)
Birth weight, g		
Mean ± SD	2877.1 ± 499.0	3062.1 ± 375.2
Birth height, cm		
Mean ± SD	48.0 ± 2.9	49.2 ± 2.0

*ART* assisted reproductive technology, *BMI* body mass index (calculated as kg/m^2^), *SD* standard deviation.

Compared with mothers who were included in the analysis (n = 60,769), those who were excluded (n = 32,172) tended to be younger, to have a lower education level, to be current smokers, to have a lower annual household income and to be multiparous.

Overall, 4912 (8.1%) of the toddlers were obese at 3 years of age using the age-specific and sex-specific BMI cut-offs (male, 17.89; female, 17.56) proposed by the International Obesity Task Force^15^. Analysis revealed that the prevalence of obesity at 3 years of age was higher in toddlers with a CS birth than in those with a vaginal birth, independent of confounders (adjusted risk ratio [aRR] 1.16, 95% confidence interval [CI] 1.08–1.25). Even when the results were analysed by sex, the risk was higher in the CS birth group in both males (aRR 1.14, 95% CI 1.01–1.27) and females (aRR 1.21, 95% CI 1.09–1.33). BMI was significantly higher in the CS birth group than in the vaginal birth group. The results are summarised in Table 2.

**Table 2:** Relationship between caesarean section birth and childhood obesity at 3 years of age according to sex (International Obesity Task Force cut-offs).

	Caesarean section	
	Yes (n = 11,241)	No (n = 49,528)
All		
Cases (BMI ≥ cut-off points), n	990	3,922
Prevalence, %	8.8	7.9
Crude risk ratio	**1.11 (1.04, 1.19)**	–
Adjusted^a^ risk ratio	**1.16 (1.08, 1.25)**	–
Adjusted^a^ body weight, M ± SEM, kg	**13.55 ± 1.49**	**13.42 ± 1.50**
Subgroup analysis		
Male		
Cases (BMI ≥ 17.89), n	417	1,666
Subtotal, n	5,716	25,320
Prevalence, %	7.3	6.6
Crude risk ratio	1.11 (0.9998, 1.23)	–
Adjusted^b^ risk ratio	**1.14 (1.01, 1.27)**	–
Adjusted^b^ body weight, M ± SEM, kg	**13.74 ± 1.45**	**13.65 ± 1.46**
Female		
Cases (BMI ≥ 17.56), n	573	2,256
Subtotal, n	5,525	24,208
Prevalence, %	10.4	9.3
Crude risk ratio	**1.11 (1.02, 1.21)**	–
Adjusted^b^ risk ratio	**1.21 (1.09, 1.33)**	–
Adjusted^b^ body weight, M ± SEM, kg	**13.35 ± 1.49**	**13.18 ± 1.51**

^a^Adjusted for maternal age, pre-pregnancy BMI, highest education level, annual household income, history of smoking, alcohol consumption, pregnancy complications, obstetric complications, history of physical disease, parity, use of assisted reproduction technology, child sex, birth term, birth weight and birth height.

^b^Adjusted for all the covariates in the model^a^, except child sex.

Bold type indicates statistical significance at the level of *p* < 0.05. – indicates reference. *BMI* body mass index.

In sensitivity analysis with obesity definitions of ≥ 16.87 for males and ≥ 17.00 for females, the prevalence of obesity was also higher in toddlers with a CS birth than in those with a vaginal birth, independent of confounders (aRR 1.10, 95% CI 1.05–1.15). However, when analysed by sex, the risk was only significant in girls (aRR 1.18, 95% CI 1.10–1.27) and not in boys (aRR 1.03, 95% CI 0.98–1.09). The results of this sensitivity analysis are summarised in Supplementary Table 1.

## Discussion

In this study, we confirmed in the Japanese population that the risk of obesity at 3 years of age is higher in children with a CS birth than in those with a vaginal birth (aRR 1.16, 95% CI 1.08–1.25) and that this finding was significant for both sexes. To the best of our knowledge, this is the first study to clarify the correlation between CS birth and childhood obesity at 3 years in a Japanese population. This correlation held even after adjusting for the many confounders that influence body habitus in childhood. In addition, our study included a very large number of participants and had sufficient statistical power to assess the correlation.

Several cohort studies have already explored the association between CS birth and obesity at 3 years of age but have yielded equivocal results^16–20^. Consistent with the present findings, cohort studies in the United States and Ireland found a positive correlation between CS birth and obesity at 3 years of age^16,17^, whereas two cohort studies in the United Kingdom found no such relationship^18,20^. Given that childhood obesity is a heterogeneous condition and the impact of CS birth on childhood obesity is not considered substantial^4,5^, some of these discordant findings could be explained by variation in the confounders in the individual studies. While all studies, including ours, have identified pre-pregnancy BMI, maternal education level, and offspring birth weight as confounders, few studies have investigated the potentially confounding effects of household income and use of assisted reproduction technology^16–20^. Furthermore, the large variations in sample size between studies (ranging from 1255 to 60,769) may have affected the accuracy of the analyses.

As a sensitivity analysis, we assessed the prevalence of childhood obesity using BMI cut-offs proposed by the Japanese Association for Human Auxology^21^, and we found that the increased risk of CS-related obesity at 3 years of age was significant only in girls. Consistent with this finding, Weng et al. identified female sex as a risk factor for obesity at 3 years of age in children with a CS birth^18^. An increased risk of sex-related health problems subsequent to CS birth has also been reported, including acute lymphoblastic leukaemia and hepatoblastoma in girls^22^ and respiratory tract infection in boys^23^. However, these differences have not been generalised, and the underlying mechanisms have not been elucidated. Moreover, this association was borderline for boys (aRR 1.03, 95% CI 0.98–1.09), and we should be cautious in concluding that there are differences according to sex.

The prevalence of obesity at 3 years of age in our study was lower than that reported in other ethnic groups^16–20^. A study that demonstrated racial and/or ethnic disparities in the prevalence of early childhood obesity suggested differences in exposure to risk factors^24^. For example, several meta-analyses have shown that maternal pre-pregnancy overweight or obesity is a distinct risk factor for childhood obesity (odds ratio [OR] 1.95 and OR 3.06, respectively)^25,26^. While 27.0% of mothers in those studies had a BMI ≥ 25, only 9.8% of those in our study had a BMI of this magnitude, which indicates that Japanese mothers are less likely to be overweight. Therefore, this study showed that the Japanese population is an ethnic group with a low prevalence of obesity in both mothers and children and provided novel evidence that CS birth is a risk factor for childhood obesity in Japanese mother–toddler pairs who tend to be nonobese. Given that Japanese individuals are vulnerable to obesity-related health problems^27^, the identification of CS birth as a risk factor for early childhood obesity in this study could be instructive from a public health perspective in Japan.

Our study has some limitations. First, we lacked information related to the underlying mechanisms by which CS birth leads to childhood obesity. Alteration of the gut microbiome is thought to be a main cause of childhood obesity in CS births, and the formation of the gut microbiome is influenced by delivery mode, preterm birth, and feeding methods up to early infancy^6,7,28^. Because we did not examine the gut microbiome directly, we adjusted for possible confounding factors that could affect gut microbiome formation. Nonetheless, potential alterations in the gut microbiome may influence the prevalence of obesity. To resolve this problem, further studies are needed to research the correlation between obesity and alteration of the gut microbiome in CS births. Second, it is not known whether children with a CS birth who are obese at 3 years of age later have adult obesity and metabolic disorders. Although adolescents with a CS birth have been shown to have a low adiponectin level and increased insulin resistance^29^, several longitudinal studies that included investigation of the relationship between childhood obesity and CS birth found that the association was no longer significant after 2–4 years of age^30,31^. Further follow-up studies are needed to determine whether CS birth-related childhood obesity truly increases the risk of future health problems.

In conclusion, the findings of this study provide new evidence that the risk of obesity at 3 years of age is higher in Japanese children with a CS birth than in those with a vaginal birth. Our results suggest that mode of delivery affects metabolic physiology and later childhood obesity, even in ethnic groups that are less prone to obesity. Further research is required to determine the mechanisms underlying the association between CS birth and childhood obesity and to identify the long-term implications for metabolic and cardiovascular health.

## Methods

### Study design and participants

**Figure 1:**
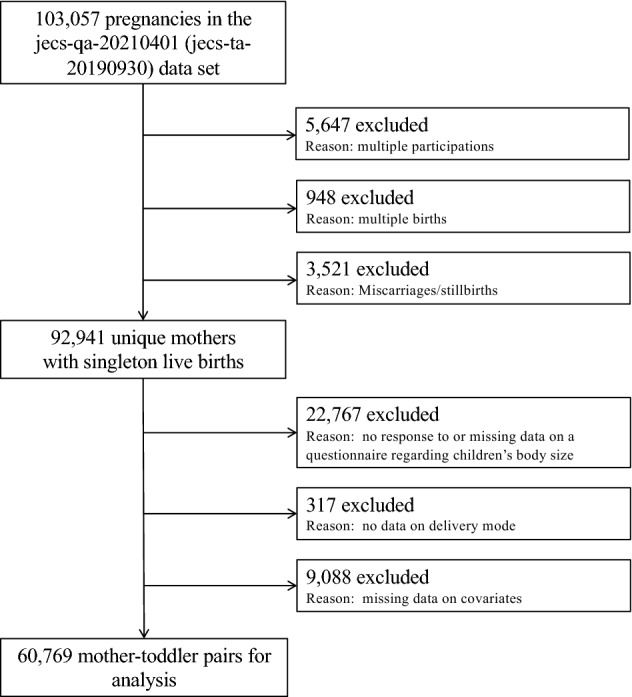
Flow diagram showing the recruitment process for pregnant women and reasons for exclusion in this study.

This study is part of a nationwide prospective birth cohort study called the Japan Environment and Children’s Study (JECS). The JECS aims to provide a foundation for policymaking and safeguard the environment for future generations. The details of its protocol have been published previously^32,33^. We decided to include only singleton children in the present study, given that anthropometric data from birth to infancy are considered to be different between multiple-birth babies and singleton babies^34^. A total of 103,057 pregnancies were registered at 15 regional centres from Hokkaido in the north to Okinawa in the south between January 2011 and March 2014. The present study analysed the *jecs-qa-20210401 (jecs-ta-20190930)* data set (released in April 2021), which includes prospectively collected data on infants at 3 years of age as well as their mothers. In this study, 5647 multiple participations (second or third registration of the same mother), 948 multiple births (twins or more) and 3511 miscarriages/still births were excluded to derive unique mothers. Among the remaining 92,941 unique mothers, 22,747 were further excluded due to lack of height or weight at 3 years of age, 317 due to lack of delivery mode and 9088 due to missing data on covariates. This left 60,769 mother–toddler pairs for complete case analysis (Fig. 1).

### Exposure

Data related to delivery mode were obtained from medical record transcripts provided by physicians, midwives/nurses, and/or research coordinators.

### Outcome

The primary outcome was obesity at 3 years of age. Because there are few children with severe obesity in Japan, we defined obesity according to the age-specific and sex-specific BMI cut-offs (males, 17.89; females, 17.56), which are the obesity cut-offs proposed by the International Obesity Task Force^15^. BMI was calculated based on the height and weight reported via questionnaires distributed and collected via mail. To minimise measurement errors, caregivers were asked to report the height and weight most recently measured at well-child visits or at hospitals and to mention the date of measurement. If unavailable, height and weight measured at nursery school or home were entered. Only birth height and weight were extracted from medical record transcripts from cooperating health care providers.

### Covariates

Body weight, smoking status, alcohol consumption, and education level are considered to be maternal factors that influence the body habitus of offspring^35–37^. Recently, it has been reported that assisted reproduction technology can affect metabolism in offspring^38,39^. Therefore, we included the following maternal covariates in the analysis: age during pregnancy, pre-pregnancy BMI, highest education level, annual household income, smoking status, alcohol consumption, pregnancy complications, obstetric complications, history of physical disease, parity and use of assisted reproduction technology (i.e., in vitro fertilisation, intracytoplasmic sperm injection, fresh/frozen embryo transfer and blastocyst transfer). The sex of the child, gestational age at birth, birth weight and birth height were included as covariates for children.

### Statistical analysis

The outcome was the number of infants with obesity at 3 years of age. The exposure variable was CS birth. Descriptive characteristics are presented as frequencies and percentages. Crude risk ratios (cRRs) and aRRs for obesity, as well as the corresponding 95% CIs, were calculated using logistic regression analysis applied to a pseudopopulation created by inverse probability of treatment weighting to reduce differences in confounders between the CS and vaginal birth groups^40,41^. The difference in BMI between the CS and vaginal birth groups was examined using a t-test applied to the pseudopopulation. When creating the pseudopopulation, all confounders were entered by the forced entry method, and birth weight and height were treated as continuous variables and included up to the quadratic term. The other variables were treated as categorical variables. In the final logistic regression analysis, cRRs and aRRs were calculated by setting the link function not to logit but to log, and CIs were calculated using robust estimation^42^. All statistical analyses were performed using SAS software (version 9.4; SAS Institute Inc., Cary, NC).

### Sensitivity analysis

We repeated the main analysis using the age-specific and sex-specific Japanese BMI cut-offs (male, 16.87; female, 17.00) proposed by the Japanese Association for Human Auxology^21^ in place of those proposed by the International Obesity Task Force^15^.

### Ethics statement

The JECS protocol was reviewed and approved by the Ministry of the Environment’s Institutional Review Board on Epidemiological Studies (No. 100910001), the Ethics Committees of all participating institutions, and the Ethics Committee of the University of Toyama (No. R2021185). All procedures contributing to the JECS comply with the ethical standards of the relevant national and institutional committees on research involving human participants and with the Helsinki Declaration of 1975, as revised in 2008, and other national regulations and guidelines. Written informed consent was obtained from all participants.

## Supplementary Information

Supplementary Table 1.

## Data Availability

Data are unsuitable for public deposition due to ethical restrictions and the legal framework of Japan. It is prohibited by the Act on the Protection of Personal Information (Act No. 57 of 30 May 2003, amendment on 9 September 2015) to publicly deposit data containing personal information. Ethical Guidelines for Medical and Health Research Involving Human Subjects enforced by the Japan Ministry of Education, Culture, Sports, Science and Technology and the Ministry of Health, Labour and Welfare also restrict the open sharing of epidemiologic data. All inquiries about access to data should be sent to: jecs-en@nies.go.jp. The person responsible for handling enquiries who can be contacted at this e-mail address is Dr Shoji F. Nakayama, JECS Programme Office, National Institute for Environmental Studies.
