# Supplementary material for: Caesarean section and childhood obesity at age 3 years derived from the Japan Environment and Children’s Study
Source: Sci Rep. 2023 Apr 21;13:6535. doi: 10.1038/s41598-023-33653-7 (PMC10121560; doi:10.1038/s41598-023-33653-7)
Supplement: Supplementary file 1 — Supplementary Table 1. [file 41598_2023_33653_MOESM1_ESM.pdf]

Supplementary Table 1. Relationship between caesarean section birth and childhood obesity at 3 years of age according to sex (Japanese Association for Human Auxology cut-offs)

	Caesarean section	
	Yes<br>(n = 11,241)	No<br>(n = 49,528)
All		
Cases, n	2,443	10,339
Prevalence, %	21.7	20.9
Crude risk ratio	**1.05 (1.001, 1.11)**	—
Adjusted[a] risk ratio	**1.13 (1.06, 1.19)**	—
Subgroup analysis		
Male		
Cases (BMI ≥ 16.87), n	1,367	5,982
Subtotal, n	5,716	25,320
Prevalence, %	23.9	23.6
Crude risk ratio	1.01 (0.96, 1.07)	—
Adjusted[b] risk ratio	1.03 (0.98, 1.09)	—
Female		
Cases (BMI ≥ 17.00), n	1,076	4,357
Subtotal, n	5,525	24,208
Prevalence, %	19.5	18
Crude risk ratio	**1.08 (1.02, 1.15)**	—
Adjusted[b] risk ratio	**1.18 (1.10, 1.27)**	—

[a]Adjusted for maternal age, pre-pregnancy BMI, highest education level, annual household income, history of smoking, alcohol consumption, pregnancy complications, obstetric complications, history of physical disease, parity, use of assisted reproduction technology, child sex, birth term, birth weight and birth height. Bold type indicates statistical significance.

[b]Adjusted for all the covariates in the model[a], except child sex.

— indicates reference. BMI, body mass index
